# Outcomes and Cost-Effectiveness of Transcatheter Versus Surgical Aortic Valve Replacement in Patients with and Without Coronary Artery Disease

**DOI:** 10.3390/jcdd12060217

**Published:** 2025-06-07

**Authors:** Horațiu Suciu, Ayman Elkahlout, Viorel Nicolae, Flavius Tomșa, Alexandru Stan, Hussam Al-Hussein, Paul-Adrian Călburean, Anda-Cristina Scurtu, David Emanuel Aniței, László Hadadi, Klara Brînzaniuc, Marius Mihai Harpa

**Affiliations:** 1 Department of Surgery IV, George Emil Palade University of Medicine, Pharmacy, Science and Technology of Targu Mures, 540142 Targu Mures, Romania; 2 Department of Cardiovascular Surgery, Emergency Institute for Cardiovascular Diseases and Transplantation, 540136 Targu Mures, Romania; 3 Department of Interventional Cardiology, Emergency Institute for Cardiovascular Diseases and Transplantation, 540136 Targu Mures, Romania; 4 Doctoral School, George Emil Palade University of Medicine, Pharmacy, Science and Technology of Targu Mures, 540142 Targu Mures, Romania; 5 Department of Anatomy and Embryology, George Emil Palade University of Medicine, Pharmacy, Science and Technology of Targu Mures, 540142 Targu Mures, Romania; 6 Department of Biostatistics and Medical Informatics, George Emil Palade University of Medicine, Pharmacy, Science and Technology of Targu Mures, 540142 Targu Mures, Romania; 7 Department of Pediatric Cardiology, Emergency Institute for Cardiovascular Diseases and Transplantation, 540136 Targu Mures, Romania

**Keywords:** transcatheter aortic valve implantation, surgical aortic valve replacement, percutaneous coronary intervention, coronary artery bypass grafting, cost-effectiveness

## Abstract

**Background:** The aim of this study was to compare costs and clinical outcomes associated with transcatheter aortic valve implantation (TAVI) versus surgical aortic valve replacement (SAVR). A secondary analysis was performed in patients with coronary artery disease, and patients with TAVI and percutaneous coronary intervention (PCI) were compared with SAVR and coronary artery bypass grafting (CABG). **Methods:** All patients who underwent the TAVI or SAVR procedure for severe degenerative aortic stenosis between August 2013 and February 2025 at a tertiary cardiovascular center were selected for inclusion in the present study. Patients were excluded if there was no available follow-up or if there was a crossover between treatments (especially CABG undergoing TAVI or SAVR undergoing PCI within a 6-month timeframe). **Results:** A total of 2452 patients (1925 undergoing SAVR and 527 undergoing TAVI) were included. Of those, 400 underwent SAVR + CABG and 75 underwent TAVI + PCI. During a median follow-up of 2.88 (1.12–6.43) years, a total of 404 all-cause events occurred, corresponding to 4.18 deaths per 100 patient-years. TAVI was associated with higher hospitalization costs and fewer in-hospital deaths than SAVR. However, long-term survival was similar between TAVI and SAVR and between TAVI + PCI and SAVR + CABG. Interventional treatment was more cost-effective in patients with EuroSCORE > 10%, while surgical treatment was more cost-effective in patients with EuroSCORE < 10%. **Conclusions:** In patients who are at high surgical risk, TAVI is more cost-effective than SAVR, and TAVI + PCI is more cost-effective than SAVR + CABG. In patients who are not at high surgical risk, SAVR is more cost-effective than TAVI, and SAVR + CABG is more cost-effective than TAVI + PCI.

## 1. Introduction

For decades, surgical aortic valve replacement (SAVR) has been the definitive therapy to relieve outflow obstruction and improve survival in severe symptomatic aortic stenosis (AS) [1]. Many patients requiring aortic valve replacement, however, are elderly or high-risk surgical candidates due to comorbidities [1]. In this context, transcatheter aortic valve implantation (TAVI) was developed as a less invasive alternative for high-risk or inoperable patients [2]. As a result, TAVI has rapidly evolved from a niche therapy into an established treatment for severe AS, now routinely considered in patients older than ~65–70 years and those with elevated surgical risk, per current guidelines [3,4].

Aortic valve stenosis commonly coexists with coronary artery disease (CAD) in this predominantly elderly patient group [5]. Due to shared risk factors, approximately half of the patients undergoing TAVI for severe AS have concomitant CAD. In the surgical era, the presence of significant CAD is managed by performing coronary artery bypass grafting (CABG) at the time of SAVR, thereby addressing both pathologies in a single operation [6]. Analogously, in patients selected for TAVI, percutaneous coronary intervention (PCI) is typically employed to revascularize significant coronary lesions either before or during the TAVI procedure [5]. Despite the high prevalence of combined AS and CAD, the most pivotal TAVI trials excluded patients with complex multi-vessel or left main CAD [7]. This exclusion has limited the generalizability of randomized trial evidence to patients with concomitant CAD, leaving the optimal management strategy in such cases uncertain [8]. In particular, it remains unclear whether a fully percutaneous approach (TAVI with PCI) yields outcomes equivalent to the traditional surgical approach (SAVR with concomitant CABG) for patients with severe AS and significant CAD [8]. Furthermore, TAVI is known to have significantly higher costs than SAVR, and it is unclear which treatment is more cost-effective, especially in intermediate- and low-risk patients.

The main aim of this study was to compare costs and clinical outcomes associated with transcatheter aortic valve implantation (TAVI) to those for surgical aortic valve replacement (SAVR). A secondary analysis was performed in patients with coronary artery disease, and patients with TAVI and percutaneous coronary intervention (PCI) were compared with those with SAVR and coronary artery bypass grafting (CABG).

## 2. Materials and Methods

The research protocol complied with the Declaration of Helsinki and was approved by the local Ethics Committees of the Emergency Institute for Cardiovascular Diseases and Transplantation Târgu Mureș (approval number 2539/04.04.2022).

All patients who underwent the TAVI or SAVR procedure for severe degenerative aortic stenosis were selected for inclusion in the present study. Exclusion criteria consisted of (1) an age less than 18 years old, (2) non-degenerative severe aortic stenosis (e.g., congenital, post-endocarditis), (3) re-intervention for pre-existing valvular prosthesis, and (4) intervention for acute cardiovascular condition. Inclusion criteria consisted of (1) SAVR performed during August 2013–February 2025, (2) TAVI performed during January 2016–February 2025, and (3) available follow-up in the Romanian National Health Insurance System database. The Romanian National Health Insurance System database supplied the mortality statuses for all the patients. For patients who died during follow-up, the Regional Statistics Office of the Romanian National Institute of Statistics supplied the exact date of death. Patients underwent interventional treatment if they were deemed at high surgical risk, defined as a EuroSCORE I ≥ 10%, a EuroSCORE II ≥ 4% (particularly after 2017 ESC Guidelines), or having additional risk factors inadequately reflected by scores, such as frailty, anemia, porcelain aorta, chest deformity, or a history of chest radiation.

Data regarding anthropometrics, relevant medical history, clinical status at hospital admission, chronic medical treatment, routine laboratory parameters, and echocardiographic parameters were collected. The laboratory parameters were the first values determined in the hospital and before the surgical or percutaneous intervention. Detailed surgical or interventional parameters were also collected. Patients with concomitant CAD were also stratified according to a combined surgical or interventional treatment (SAVR + CABG versus TAVI + PCI). While SAVR and CABG were always performed simultaneously, PCI and TAVI were considered together if performed within a 6-month timeframe. An additional exclusion criterion was considered if patients had treatment crossover (especially CABG undergoing TAVI or SAVR undergoing PCI within a 6-month timeframe). Electronic health records provided detailed hospitalization costs.

A significance level α of 0.05 and a 95% confidence interval (CI) were considered. Continuous variables were evaluated for normal distribution using the Shapiro–Wilk test. Continuous variables with parametric distributions were reported as mean ± standard deviation and compared using a non-paired or paired Student *t*-test, while continuous variables with non-parametric distributions and discrete variables were reported as median (interquartile range) values and compared using the Mann–Whitney or Wilcoxon test. Categorical variables were reported as absolute and relative frequencies and compared using the Fisher exact test. The survival curves were assessed using the Kaplan–Meier method and compared using the log-rank test. A 1:1 nearest-neighbor matching test on the continuous variables was performed without replacement, matching each patient in the smaller cohort to the closest-score patient in the larger cohort to yield balanced TAVI and SAVR groups. To examine the impacts of different predictors on survival, univariable and multivariable Cox proportional hazard models were used to predict the association in the form of a hazard ratio (HR) between observed survival and single or multiple independent variables, respectively. The number of predictors in the multivariable models was chosen so that there would be at least 15 events per covariate [9]. Multicollinearity among independent variables in multivariable models was assessed, and a strong correlation between variables was considered present if the variance inflation factor was above 2.5. Multivariable models were built via forward stepwise selection based on AIC. At each iteration, the predictor whose inclusion most reduced the AIC was retained, and the process continued until no remaining candidate yielded a lower AIC. AIC’s built-in penalty for parameter count provided protection against overfitting. Group-specific mean costs were calculated and restricted mean survival time (RMST) was obtained by integrating Kaplan–Meier curves with censoring up to the maximum follow-up; cost per RMST day was then derived and the ICER computed as the difference in mean cost divided by the difference in RMST between TAVI and SAVR. Statistical analysis was performed using R version 4.1.1 and R Studio version 1.4.17. Figures were plotted using matplotlib and lifelines packages in Python 4.9.13.

## 3. Results

A total of 2452 patients were included, of which 1423 (58.0) were males, with a median age of 69 (62–74) years. In total, 1925 (78.5%) patients underwent SAVR, of which 404 (20.9%) underwent combined SAVR + CABG. Moreover, 527 (21.5%) underwent TAVI, of which 75 (14.2%) underwent combined TAVI + PCI. The complete baseline characteristics of the included population are reported in Table 1. The complete baseline characteristics of the TAVI + PCI and SAVR + CABG groups are reported in Supplemental Table S1.

Patients who underwent TAVI had higher age and higher incidence of stroke, diabetes mellitus, atrial fibrillation, and chronic obstructive pulmonary disease (COPD). TAVI patients had a lower left ventricular ejection fraction (LVEF) than SAVR patients and had higher creatinine and lower hemoglobin levels. In-hospital death occurred in 101 (4.1%) patients. In-hospital death was higher in surgically treated patients than in intervention-treated patients (Table 2).

**Figure 1:**
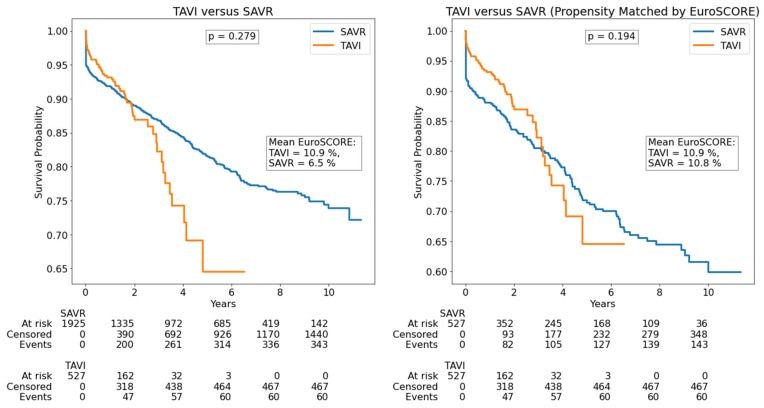
Survival among severe aortic stenosis patients. SAVR—surgical aortic valve replacement; TAVI—transcatheter aortic valve implantation.

During a median follow-up of 2.88 (1.12–6.43) years, a total of 404 all-cause events occurred, corresponding to 4.18 deaths per 100 patient-years. All-cause TAVI deaths occurred in 7.25 cases per 100 patient-years, while all-cause SAVR deaths occurred in 3.89 cases per 100 patient-years. Survival curves for patients with severe degenerative aortic stenosis, including propensity matched by EuroSCORE, are illustrated in Figure 1. Propensity matching by age is reported in Supplemental Figure S1.

**Figure 2:**
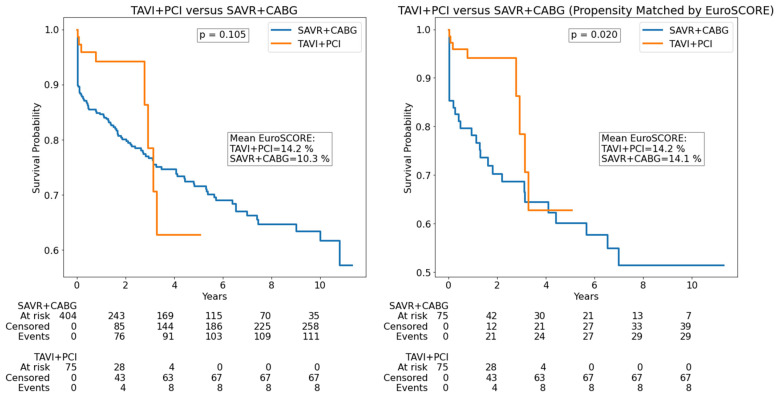
Survival among severe aortic stenosis patients with concomitant coronary artery disease (excluding patients without coronary artery disease). CABG—coronary artery bypass grafting; PCI—percutaneous coronary intervention; SAVR—surgical aortic valve replacement; TAVI—transcatheter aortic valve implantation.

**Figure 3:**
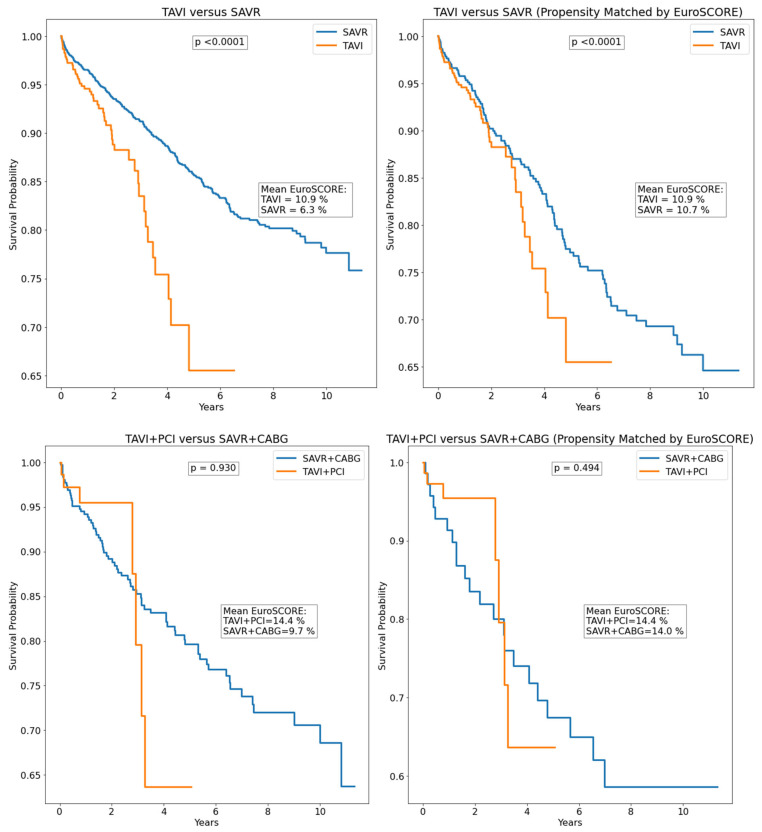
Out-of-hospital survival in the studied population (excluding in-hospital deaths). CABG—coronary artery bypass grafting; PCI—percutaneous coronary intervention; SAVR—surgical aortic valve replacement; TAVI—transcatheter aortic valve implantation.

There was no survival difference in TAVI versus SAVR patients, including in EuroSCORE-matched groups; however, after propensity matching by age, TAVI patients had higher survival than SAVR patients. Survival curves for patients with severe degenerative aortic stenosis and coronary artery disease, including propensity matched by EuroSCORE, are illustrated in Figure 2. Propensity matching by age is reported in Supplemental Figure S2. There was no survival difference in TAVI + PCI versus SAVR + CABG patients; however, after propensity matching by both EuroSCORE and age, TAVI + PCI patients had higher survival than SAVR + CABG patients. Since SAVR is known to be associated with higher in-hospital mortality (due to higher intraoperative death, which is its main limitation in comparison to TAVI), a separate analysis was performed on out-of-hospital death. After excluding in-hospital deaths, there was no survival difference in surgically versus intervention-treated patients (Figure 3). However, after propensity matching for EuroSCORE, SAVR had lower mortality than TAVI in out-of-hospital death. In the multivariable Cox analysis, age, creatinine, hemoglobin, LVEF, COPD, atrial fibrillation, and stroke were independent predictors of mortality, while there was no difference between surgical and interventional treatment (Table 3).

Regarding medical therapy, beta-blockers were prescribed in 1720 (70.1%) patients, angiotensin-converting-enzyme inhibitors or angiotensin receptor blockers were prescribed in 1699 (69.2%) patients, angiotensin receptor–neprilysin inhibitor therapy was used in 101 (4.1%) patients, sodium-glucose co-transporter 2 was used in 95 (3.8%) patients, statin therapy was used in 2201 (89.7%) patients, and aspirin was used in 1989 (81.1%) patients. There was no heterogeneity among groups regarding the medical treatment. There was no significant impact of the medical treatment on patient survival.

**Figure 4:**
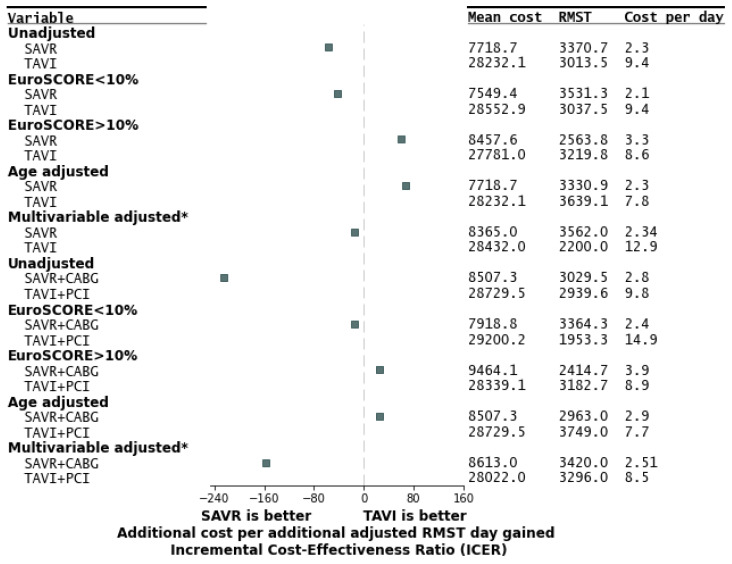
A forest plot with the cost-effectiveness of SAVR versus TAVI. CABG—coronary artery bypass graft; PCI—percutaneous coronary intervention; RMST—restricted mean survival time; SAVR—surgical aortic valve replacement; TAVI—transcatheter aortic valve implantation. * Adjusted for age, hemoglobin, creatinine, LVEF, stroke, COPD, and AF.

In the cost-effectiveness analysis, in the unadjusted SAVR versus TAVI comparison, SAVR was more cost-effective than TAVI (mean cost of EUR 7718 versus UER 28,232, additional cost per restricted mean survival time [RMST] day gained of EUR −57.4/day, favoring SAVR; Figure 4). When adjusting for age, TAVI was more cost-effective than SAVR (additional cost per RMST day gained of EUR 66.6/day). When adjusting for multiple clinical variables, SAVR was slightly more cost-effective than TAVI (additional cost per RMST day gained of EUR −14.7 /day). Similarly, SAVR + CABG was more cost-effective than TAVI + PCI (mean cost of EUR 8507 versus EUR 28,729, additional cost per restricted mean survival time [RMST] day gained of EUR −224.9/day; Figure 4). When adjusting for age, TAVI + PCI was more cost-effective than SAVR + CABG (additional cost per RMST day gained of EUR 25.7/day). When adjusting for multiple clinical variables, SAVR + CABG was slightly more cost-effective than TAVI + PCI (additional cost per RMST day gained of EUR −156.5/day).

Furthermore, when stratifying according to EuroSCORE, surgical treatment was more cost-effective in non-high-risk patients, and interventional treatment was more cost-effective in high-risk patients. In patients with EuroSCORE < 10%, SAVR versus TAVI had an additional cost per RMST day gained of EUR −42.5/day (favoring SAVR), while SAVR + CABG versus TAVI + PCI had an additional cost per RMST day gained of EUR −15.8/day (favoring SAVR + CABG). In patients with EuroSCORE > 10%, SAVR versus TAVI had an additional cost per RMST day gained of EUR 59.5/day (favoring TAVI), while SAVR + CABG versus TAVI + PCI had an additional cost per RMST day gained of EUR 24.5/day (favoring TAVI + PCI).

## 4. Discussion

The main findings of this study are as follows: (1) TAVI patients were older and had a higher burden of associated comorbidities such as atrial fibrillation, stroke, and COPD than SAVR. (2) TAVI was associated with a higher hospitalization cost, lower hospitalization length, and lower in-hospital death rate than SAVR. (3) There was no survival difference between TAVI and SAVR. Similarly, there was no survival difference between TAVI + PCI and SAVR + CABG. When considering out-of-hospital survival, surgical treatment had better long-term outcomes when compared with interventional treatment. (4) In patients with high surgical risk given by a EuroSCORE above 10%, TAVI was more cost-effective than SAVR, and TAVI + PCI was more cost-effective than SAVR + CABG. However, in patients without high surgical risk, given by a EuroSCORE below 10%, SAVR was more cost-effective than TAVI, and SAVR + CABG was more cost-effective than TAVI + PCI.

Our findings are consistent with a growing body of literature comparing transcatheter and surgical valve replacement outcomes. Numerous prior studies have reported lower perioperative mortality and shorter hospitalization with TAVI compared to SAVR, particularly in elderly or high-risk populations [10]. For example, a large propensity-matched analysis of octogenarians found in-hospital mortality of 3.4% with TAVI vs. 6.8% with SAVR, along with a reduced hospitalization length in the TAVI group [10]. These data underscore the immediate safety advantage and faster recovery associated with transcatheter therapy. Our observation of no significant difference in adjusted long-term survival between TAVI and SAVR is also in line with evidence from randomized trials. A recent meta-analysis of seven trials with up to ~5 years follow-up showed no significant differences in all-cause mortality or disabling stroke between TAVI and surgery, indicating equivalent mid-term survival outcomes [11]. Thus, our study’s long-term survival findings reinforce the consensus that TAVI offers similar longevity to SAVR at least through mid-term follow-up, once differences in patient risk profiles are accounted for.

In patients with combined severe aortic stenosis and coronary artery disease, there are less published data, but emerging studies support our results. In a recent multicenter registry analysis, the one-year composite rate of death or stroke was significantly lower in patients treated with TAVI + PCI compared to SAVR + CABG (4.5% vs. 8.8% in a propensity-matched comparison) [12]. This suggests that a complete percutaneous approach can confer an early outcome advantage, even when accounting for baseline risk differences (indeed, the TAVI + PCI patients in that study were older and of higher risk). A systematic review and meta-analysis by Tarus et al. similarly found that TAVI with PCI yields certain early benefits over SAVR with CABG, including less major bleeding and shorter hospital stays [13]. However, that meta-analysis also highlighted important caveats: TAVI + PCI was associated with a higher need for pacemaker implantation and more frequent coronary re-interventions during follow-up, and long-term survival was modestly lower in the TAVI + PCI group relative to SAVR + CABG. The reduced long-term survival in transcatheter-treated patients likely reflects residual differences in patient comorbidities and the possibility of less complete revascularization with PCI. Overall, our results are in accordance with these studies by showing a clear short-term benefit to the percutaneous strategy, while long-term outcomes may converge or even favor surgery using certain metrics. Taken together with the literature, our study supports the view that transcatheter approaches are an attractive option for high-risk patients or those who may not tolerate open surgery well, whereas surgery with concomitant CABG may still benefit certain patients by ensuring durable valve and coronary results. Furthermore, patients with concomitant valvular and CAD had high long-term mortality rates, with survival rates after 5 years being approximately 75%. Those findings are in line with our previous reports stating that Eastern European populations have a particularly long-term mortality rate [14,15]. Interestingly, the medical treatment did not influence survival rates in our study. It is known that pharmacological treatment does not affect mortality in severe aortic stenosis, except for SGLT2 inhibitors, possibly due to their multiple pleiotropic effects [16,17]. In our study, the rate of SGLT2 inhibitor usage was low, possibly due to a lack of a nationwide compensation scheme.

From a health economics perspective, our finding that TAVI was more cost-effective in high-risk patients and SAVR more cost-effective in low-risk patients aligns with prior analyses of cost and value in aortic stenosis treatment. Early economic studies established that TAVI provides good value in patients who are inoperable or at high surgical risk, given the significant gains in survival and quality of life compared to medical therapy or high-risk surgery [18]. In intermediate-risk cohorts, model-based analyses (incorporating trial data) have also found TAVI to be cost-effective, with incremental cost-effectiveness ratios (ICERs) falling under conventional willingness-to-pay thresholds in many healthcare systems [18]. For instance, one analysis using PARTNER trial data reported an ICER around USD ~46,000 per quality-adjusted life year (QALY) for TAVI versus SAVR in intermediate-risk patients, which is within the accepted range for cost-effective care. These analyses build on the premise that although TAVI has higher device and procedural costs, it can offset some costs by reducing ICU/hospital days and by improving survival or quality of life. Indeed, in our study and others, the index hospitalization cost for TAVI is consistently higher than that for SAVR, but TAVI patients may accumulate more QALYs over time due to slightly better early outcomes or the avoidance of very-high-risk surgery. Crucially, the cost-effectiveness of TAVI depends on the patient risk profile and device costs. Our finding that TAVI was not cost-effective in low-risk patients is supported by the notion that when surgical risk is low, the incremental benefit of TAVI is minimal, while its extra costs are substantial. In lower-risk (often younger) patients, SAVR has excellent outcomes and low operative mortality, so the high price of transcatheter valves may not be justified unless TAVI demonstrates a clear improvement in outcomes. Some recent studies have even suggested that TAVI can be cost-effective in low-risk populations under certain conditions [19]—for example, a Danish analysis of the NOTION trial projected TAVI to be cost-effective in a low-risk cohort over a lifetime horizon, albeit with a very small QALY gain and assuming favorable long-term results. However, those conclusions are highly sensitive to assumptions about valve durability, complication rates, and device pricing. If a transcatheter valve does not last as long and requires re-intervention in a younger patient, the cost-effectiveness would worsen. An analysis from the PARTNER 2, which included intermediate-risk patients, showed that while procedural costs were about USD ~20,000 higher for TAVI than SAVR, the total cost of the index hospitalization was similar between TAVI and SAVR [20]. In contrast, in our study, TAVI hospitalizations were over EUR 20,000 more expensive than SAVR; the difference in procedural costs was also reflected in total hospitalization cost. Our results, in agreement with most of the literature, emphasize that while TAVI is a valuable and even dominant strategy for high-risk patients, its economic advantage diminishes as patient risk decreases. Therefore, the existing literature supports a selective approach: using TAVI where it confers a clear net benefit and continuing to utilize SAVR in low- and intermediate-risk patients until further evidence (e.g., longer-term outcomes and lower device costs) can justify the broader use of TAVI in that group.

The main limitation of this study was its retrospective nature. Also, our study was a non-randomized observational study, which inherently carries a risk of residual confounding. Also, we used EuroSCORE I instead of EuroSCORE II since the latter was not available for all the included patients. We employed multivariate adjustment to account for baseline differences, but unmeasured factors (e.g., frailty, calcification pattern) could have influenced outcomes. Our cost and cost-effectiveness findings are subject to the cost structures and pricing in our healthcare context, which may differ in other countries or systems. We did not include indirect costs (such as rehabilitation, long-term care, or loss of productivity) and focused on direct medical costs. Despite these limitations, this study draws strength from a large, real-world cohort and reflects contemporary practice outcomes.

## 5. Conclusions

In patients with high surgical risk, TAVI is more cost-effective than SAVR, and TAVI + PCI is more cost-effective than SAVR + CABG. This benefit is linked to lower procedural and in-hospital death with interventional treatment (TAVI or TAVI + PCI) in high-risk patients. However, in patients without high surgical risk, SAVR is more cost-effective than TAVI, and SAVR + CABG is more cost-effective than TAVI + PCI. While other studies showed similar hospitalization costs between TAVI and SAVR, in our study, TAVI hospitalization costs were over EUR 20,000 higher than those for SAVR, since the difference in procedural costs was also maintained in the total hospitalization cost.

## Figures and Tables

**Table 1:** The complete clinical characteristics of the studied population.

Parameter	All Patients (n = 2452)	SAVR (n = 1925)	TAVI (n = 527)	*p*
Age (years)	68.5 (61.6–73.8)	66.4(59.1–71.1)	77.3(73.3–81.1)	<0.0001
Male sex	1423 (58.03%)	1134 (58.91%)	289 (54.84%)	0.10
Cost (EUR ×1000)	8.42(4.68–15.54)	6.64(4.18–9.69)	28.00(26.17–29.93)	<0.0001
Hospitalization length (days)	10 (8–14)	11 (9–14)	7 (5–10)	<0.0001
Hypercholesterolemia	1021 (41.64%)	761 (39.53%)	260 (49.34%)	0.0005
Diabetes mellitus	757 (30.87%)	557 (28.94%)	200 (37.95%)	0.001
HBP	1923 (78.43%)	1464 (76.05%)	459 (87.1%)	0.0005
Atrial fibrillation	707 (28.83%)	509 (26.44%)	198 (37.57%)	0.0005
CKD	135 (5.51%)	55 (2.86%)	80 (15.18%)	0.0005
History of stroke	110 (4.49%)	70 (3.64%)	40 (7.59%)	0.001
History of MI	344 (14.03%)	237 (12.31%)	107 (20.3%)	0.0005
LBBB	141 (5.75%)	51 (2.65%)	90 (17.08%)	0.0005
Smoker	143 (5.83%)	126 (6.55%)	17 (3.23%)	0.0025
COPD	122 (4.98%)	83 (4.31%)	39 (7.4%)	0.005
DCM	115 (4.69%)	75 (3.9%)	40 (7.59%)	0.0005
CAD	537 (21.9%)	415 (21.5%)	122 (23.1%)	0.73
PAD	167 (6.8%)	103 (5.3%)	64 (12.1%)	<0.0001
Creatinine (mg/dL)	1.01(0.82–1.27)	1 (0.8–1.25)	1.05 (0.85–1.31)	0.02
Hemoglobin (×10^3^/µL)	13.2(12–14.2)	13.3 (12.1–14.28)	12.77 ± 1.68	<0.0001
Platelets (×10^3^/µL)	206 (172–247)	206 (173–247)	205 (170- 246)	0.60
Leucocytes (×10^3^/µL)	7.13 (5.99–8.42)	7.19 (6–8.45)	6.98 (5.89–8.26)	0.24
LVEF (%)	55 (50–60)	55 (50–60)	50 (45–50)	0.0001
Mean AV gradient (mmHg)	46.2 (39.9–53.2)	48.5 (42.8–57)	44.1 (39.7–52.9)	0.11
Maximum AV velocity (m/s)	4.31 (3.84–5.12)	4.49 (4–5.33)	4.27 (3.8–5.07)	0.09
Mean AVA (cm^2^)	0.79 (0.71–0.99)	0.75 (0.67–0.86)	0.82 (0.72–0.92)	0.04
Post-procedure mean AV gradient (mmHg)	11.9 (10.4–13.9)	12.2 (10.4–13.9)	11.5 (9.8–13.1)	0.75
Low-flow low-gradient severe AS	111 (4.5%)	86 (1.1%)	25 (4.1%)	0.87
EuroSCORE (%)	8.72 ± 7.82	6.44 ± 5.82	10.94 ± 8.12	<0.0001
EuroSCORE > 10%	799 (32.5%)	360 (18.7%)	439 (83.3%)	<0.0001

CAD—coronary artery disease; CKD—chronic kidney disease; COPD—chronic obstructive pulmonary disease; DCM -dilated cardiomyopathy; HBP—high blood pressure; LBBB—left bundle branch block; MI—myocardial infarction; SAVR—surgical aortic valve replacement; TAVI—transcatheter aortic valve implantation.

**Table 2:** Complications among studied groups.

**Complication**	**SAVR (n = 1925)**	**TAVI (n = 527)**	***p***
Stroke	9 (0.6%)	2 (0.3%)	0.78
Tamponade	7 (0.3%)	2 (0.3%)	0.95
Acute kidney injury	77 (4.0%)	8 (1.5%)	0.008
Dialysis	27 (1.4%)	2 (0.3%)	0.08
Bleeding requiring transfusion *	266 (13.8%)	26 (4.9%)	<0.0001
Pacemaker implantation **	40 (2.0%)	55 (10.4%)	<0.0001
In-hospital mortality	93 (4.8%)	8 (1.5%)	0.001
**Complication**	**SAVR + CABG (n = 327)**	**TAVI + PCI (n = 75)**	***p***
Stroke	8 (2.1%)	1 (1.3%)	0.99
Tamponade	3 (0.9%)	1 (1.3%)	0.56
Acute kidney injury	52 (15.9%)	4 (5.3%)	0.01
Dialysis	5 (1.5%)	1 (1.3%)	0.99
Bleeding requiring transfusion *	67 (20.4%)	3 (4.0%)	0.0003
Pacemaker implantation **	9 (2.7%)	9 (12.0%)	0.002
In-hospital mortality	26 (7.9%)	1 (1.3%)	0.03

CABG—coronary artery bypass grafting; PCI—percutaneous coronary intervention; TAVI—transcatheter aortic valve implantation; SAVR—surgical aortic valve replacement. * Including intraoperative blood transfusion. ** Pacemaker implantation was considered as de novo implantation within 30 days following intervention.

**Table 3:** Univariable and multivariable Cox regression for predicting survival after TAVI or SAVR in the entire population of this study.

Parameter	Univariate Cox Regression	Stepwise Multivariate Cox Regression
Age (years)	*p* < 0.001, HR = 1.05 (1.03–1.06)	*p* < 0.001, HR = 1.04 (1.03–1.06)
Male sex	*p* = NS	-
Hypercholesterolemia	*p* = 0.05, HR = 0.81 (0.66–1.00)	-
Diabetes mellitus	*p* < 0.001, HR = 1.63 (1.33–1.99)	-
HBP	*p* = 0.02, HR = 1.33 (1.04–1.70)	-
Atrial fibrillation	*p* < 0.001, HR = 1.64 (1.34–2.02)	*p* = 0.01, HR = 1.45 (1.09–1.93)
CKD	*p* < 0.001, HR = 2.28 (1.59–3.29)	-
History of stroke	*p* < 0.001, HR = 1.90 (1.28–2.83)	*p* < 0.001, HR = 2.35 (1.47–3.77)
History of MI	*p* = 0.04, HR = 1.34 (1.01–1.78)	-
LBBB	*p* = NS	-
Smoker	*p* = NS	-
COPD	*p* = 0.02, HR = 1.57 (1.06–2.32)	*p* = 0.02, HR = 1.74 (1.1–2.75)
DCM	*p* = NS	-
Number of CAD	*p* = 0.05, HR = 1.17 (1.00–1.37)	-
LVEF (%)	*p* < 0.001, HR = 0.97 (0.96–0.99)	*p* = 0.02, HR = 0.98 (0.97–1.00)
Creatinine (mg/dL)	*p* < 0.001, HR = 1.52 (1.28–1.81)	*p* < 0.001, HR = 1.05 (1.04–1.08)
Hemoglobin (×10^3^/µL)	*p* = 0.01, HR = 1.08 (1.02–1.14)	*p* = 0.02, HR = 1.08 (1.01–1.16)
Platelets (×10^3^/µL)	*p* = NS	-
Leucocytes (×10^3^/µL)	*p* = NS	-
TAVI performed *	*p* = 0.03, HR = 0.89 (0.82–0.97)	-
PCI performed	*p* < 0.001, HR = 2.89 (1.43–5.84)	-
CABG performed	*p* < 0.001, HR = 1.63 (1.28–2.07)	-
EuroSCORE (%)	*p* < 0.001, HR = 1.056 (1.046–1.067)	- **

CABG—coronary artery bypass graft; CAD—coronary artery disease; CKD—chronic kidney disease; COPD—chronic obstructive pulmonary disease; DCM—dilated cardiomyopathy; HBP—high blood pressure; LBBB—left bundle branch block; MI—myocardial infarction; NS—not significant; PCI—percutaneous coronary intervention; SAVR—surgical aortic valve replacement; TAVI—transcatheter aortic valve implantation. * TAVI performed instead of SAVR. ** Excluded from multivariable analysis due to partial overlapping with other parameters (e.g., LVEF, age).

## Data Availability

Due to national and EU regulations, particularly the General Data Protection Regulation (GDPR), the data used in this study cannot be made publicly available and shared with the wider research community. However, the data can be shared by the corresponding author for use in secure environments upon reasonable request.

## Supplementary Materials

The following supporting information can be downloaded at https://www.mdpi.com/article/10.3390/jcdd12060217/s1. Table S1—Complete clinical characteristics of the studied population. Figure S1—Survival among severe aortic stenosis patients. Figure S2—Survival among severe aortic stenosis with coronary artery disease patients. Figure S3—Out-of-hospital survival in the studied population (excluding in-hospital deaths).
