# Supplementary material for: Outcomes and Cost-Effectiveness of Transcatheter Versus Surgical Aortic Valve Replacement in Patients with and Without Coronary Artery Disease
Source: J Cardiovasc Dev Dis. 2025 Jun 7;12(6):217. doi: 10.3390/jcdd12060217 (PMC12193443; doi:10.3390/jcdd12060217)
Supplement: Supplementary file 1 [file jcdd-12-00217-s001.zip › jcdd-3596222-supplementary.pdf]

Supplemental Table S1 – Complete clinical characteristics of the studied population.

Parameter	All patients (n=479)	TAVI+PCI (n=75)	SAVR+CABG (n=404)	p
Age (years)	69 (62 - 73)	78 (75 - 81)	67 (62 - 72)	<0.0001
Male sex	326 (68.06%)	43 (57.33%)	283 (70.05%)	0.03
Cost (x1000 €)	8.40 (4.96 – 13.87)	28.93 (26.72 – 30.99)	7.18 (4.58 – 10.61)	<0.0001
Hospitalization length (days)	11 (8 - 14)	8 (5 - 11)	12 (9 - 15)	<0.0001
Hypercholesterolemia	63 (13.15%)	3 (4%)	60 (14.85%)	0.01
HBP	230 (48.02%)	33 (44%)	197 (48.76%)	0.46
Atrial fibrillation	36 (7.53%)	6 (8%)	29 (7.17%)	0.01
CKD	117 (24.43%)	23 (30.67%)	94 (23.27%)	0.18
History of stroke	35 (7.31%)	10 (13.33%)	25 (6.19%)	0.03
History of MI	43 (8.98%)	6 (8%)	37 (9.16%)	0.83
LBBB	21 (4.38%)	1 (1.33%)	20 (4.95%)	0.22
Smoker	39 (8.14%)	15 (20%)	24 (5.94%)	<0.0001
DCM	24 (5.01%)	8 (10.67%)	16 (3.96%)	0.02
Creatinine (mg/dl)	1.04 (0.88 - 1.44)	1.02 (0.84 - 1.42)	1.04 (0.9 - 1.48)	0.20
Hemoglobin($\times10^3/\mu$L)	13.2 (12 - 14.2)	12.59 ± 1.55	13.3 (12.1 - 14.2)	0.006
Platelets ($\times10^3/\mu$L)	209 (173 - 249.75)	202 (162 - 243)	210 (175 - 250)	0.49
Leucocytes ($\times10^3/\mu$L)	7.28 (6.13 - 8.55)	6.83 (6.01 - 8.26)	7.37 (6.24 - 8.62)	0.14
LVEF (%)	55 (50-60)	45 (45-50)	55 (50-60)	0.0001
EuroSCORE (%)	8.3 (5.1 - 13.6)	11.01 (7.4 - 16.1)	7.6 (4.6 - 13.2)	<0.0001
EuroSCORE >10%	195 (40.71%)	41 (54.67%)	154 (38.12%)	0.01
Number of diseased vessels	2 (1 - 3)	2 (1 - 3)	3 (2 - 3)	<0.0001
Left main disease	35 (7.30%)	5 (6.66%)	30 (7.42%)	0.99
Number of vein grafts	-	-	2 (1 - 2)	-
Number of arterial grafts	-	-	0 (0 - 1)	-
Only venous grafts	-	-	360 (89.1%)	-
Only arterial grafts	-	-	7 (1.73%)	-
Mixed grafts	-	-	37 (9.15%)	-
Number of stents	-	2 (2 - 3)	-	-
Length of stent (mm)	-	27 (23 - 32)	-	-

CAD – coronary artery disease; CKD – chronic kidney disease; COPD – chronic obstructive pulmonary disease; DCM -dilated cardiomyopathy; HBP – high blood pressure; LBBB – left bundle branch block; MI – myocardial infarction; SAVR – surgical aortic valve replacement; TAVI – transcatheter aortic valve implantation.

Supplemental Figure S1 – Survival among severe aortic stenosis patients.

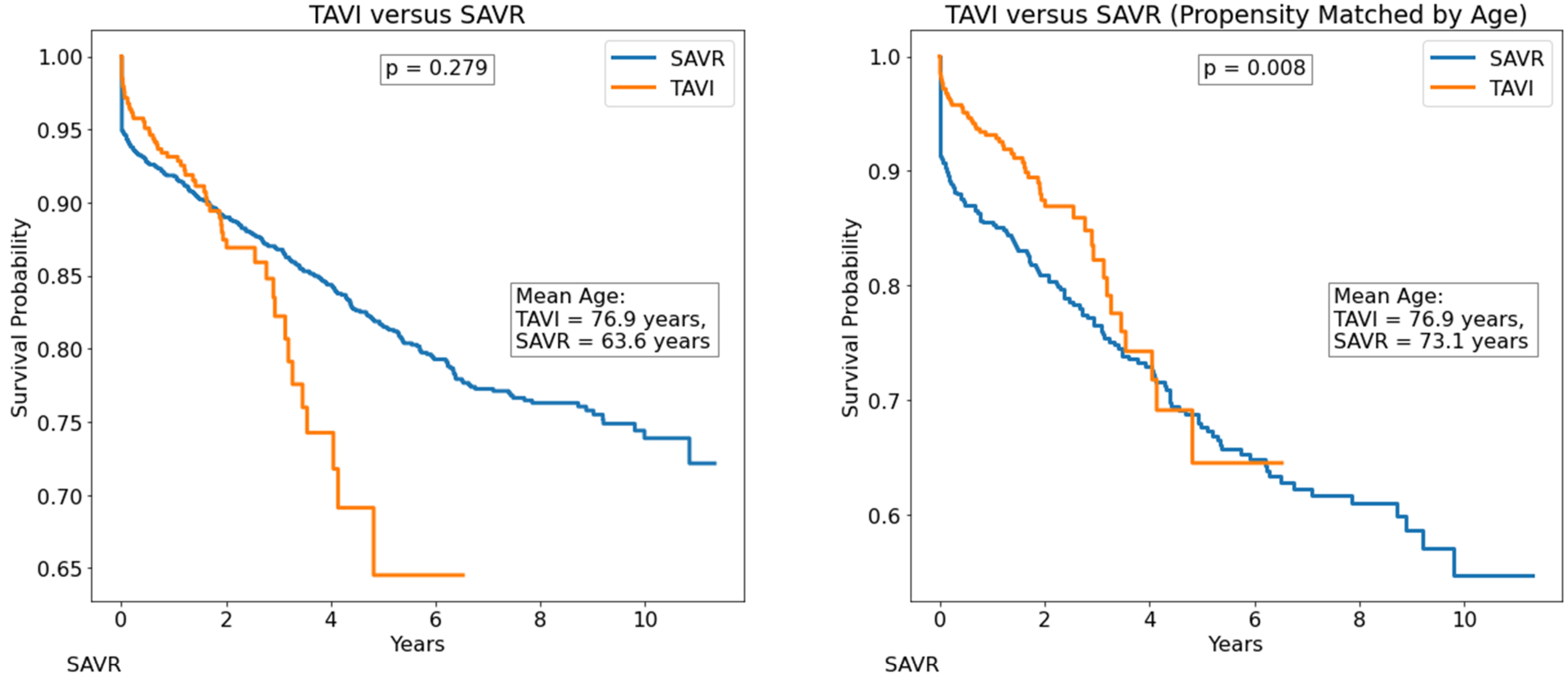

TAVI versus SAVR

SAVR	0	2	4	6	8	10
At risk	1925	1335	972	685	419	142
Censored	0	390	692	926	1170	1440
Events	0	200	261	314	336	343

TAVI	0	2	4	6	8	10
At risk	527	162	32	3	0	0
Censored	0	318	438	464	467	467
Events	0	47	57	60	60	60

TAVI versus SAVR (Propensity Matched by Age)

SAVR	0	2	4	6	8	10
At risk	527	331	217	140	79	21
Censored	0	101	187	242	296	350
Events	0	95	123	145	152	156

TAVI	0	2	4	6	8	10
At risk	527	162	32	3	0	0
Censored	0	318	438	464	467	467
Events	0	47	57	60	60	60

SAVR – surgical aortic valve replacement; TAVI – transcatheter aortic valve implantation.

Supplemental Figure S2 – Survival among severe aortic stenosis with coronary artery disease patients.

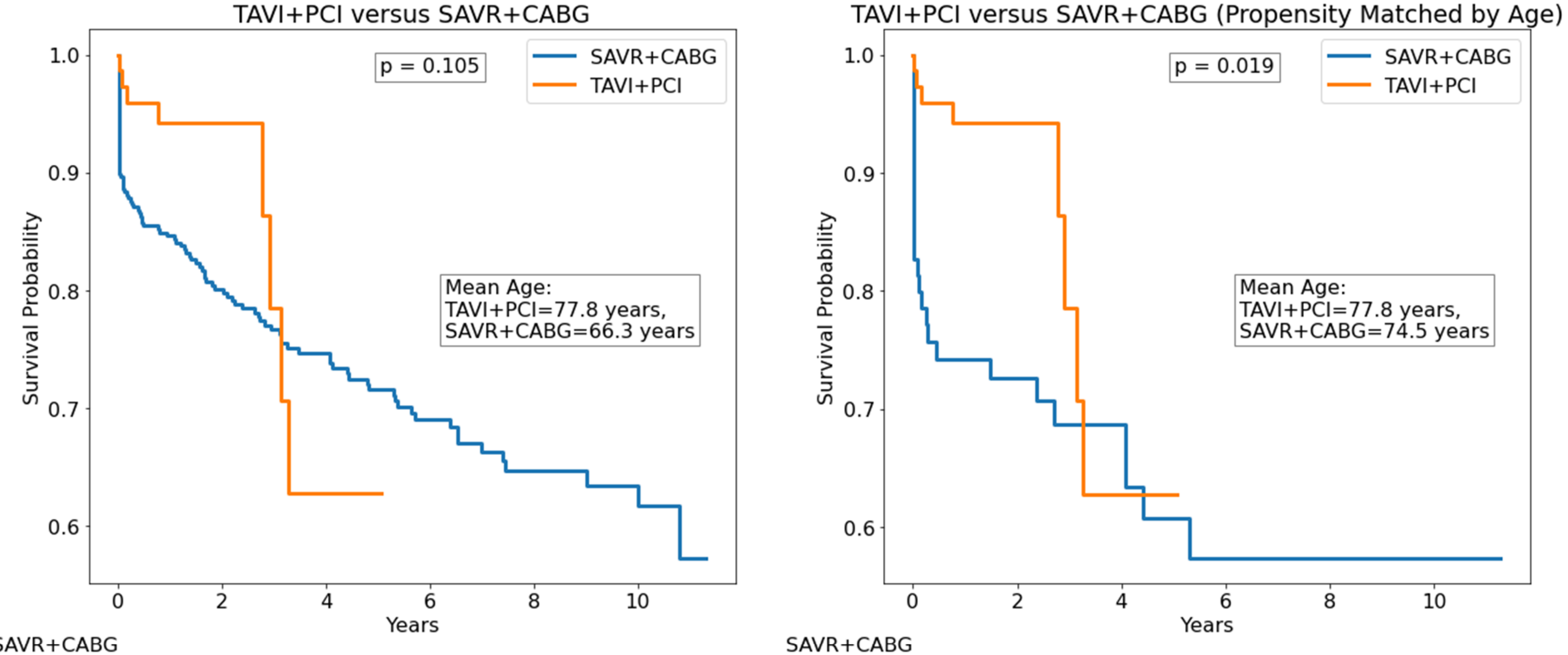

TAVI+PCI versus SAVR+CABG

SAVR+CABG	0	2	4	6	8	10
At risk	404	243	169	115	70	35
Censored	0	85	144	186	225	258
Events	0	76	91	103	109	111

TAVI+PCI	0	2	4	6	8	10
At risk	75	28	4	0	0	0
Censored	0	43	63	67	67	67
Events	0	4	8	8	8	8

TAVI+PCI versus SAVR+CABG (Propensity Matched by Age)

SAVR+CABG	0	2	4	6	8	10
At risk	75	41	26	15	10	4
Censored	0	14	27	34	39	45
Events	0	20	22	26	26	26

TAVI+PCI	0	2	4	6	8	10
At risk	75	28	4	0	0	0
Censored	0	43	63	67	67	67
Events	0	4	8	8	8	8

CABG - coronary artery bypass grafting; PCI – percutaneous coronary intervention; SAVR – surgical aortic valve replacement; TAVI – transcatheter aortic valve implantation.

Supplemental Figure S3 – Out-of-hospital survival in the studied population (excluding in-hospital deaths).

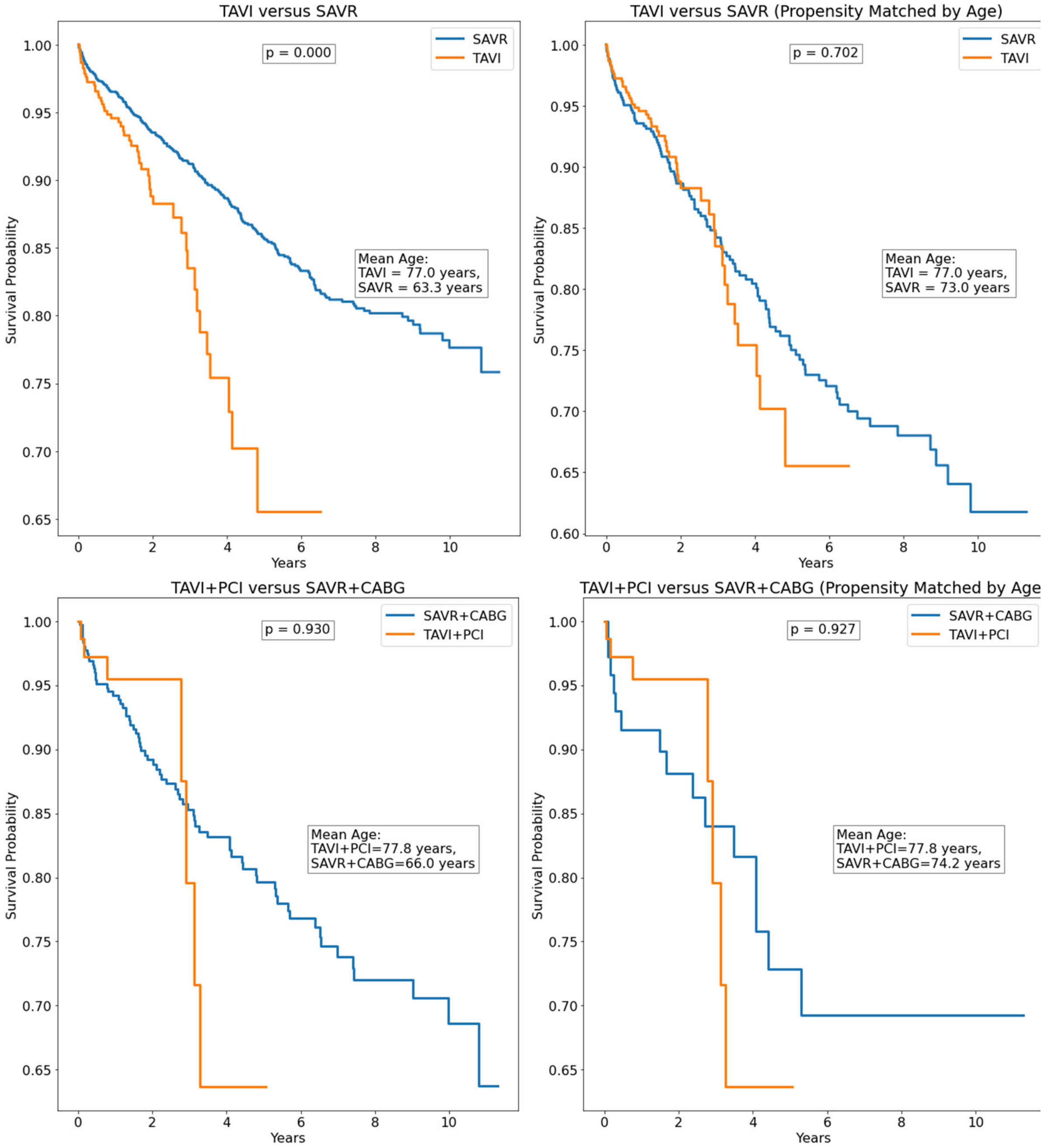

CABG - coronary artery bypass grafting; PCI – percutaneous coronary intervention; SAVR – surgical aortic valve replacement; TAVI – transcatheter aortic valve implantation.
